# Supplementary figures and images for: Asymmetric envelope surface disposition of secreted protein YjbI controls bimodal antibiotic susceptibilities in C. crescentus
Source: EMBO J. 2026 Jan 3;45(3):987–1023. doi: 10.1038/s44318-025-00668-x (PMC12864828; doi:10.1038/s44318-025-00668-x)

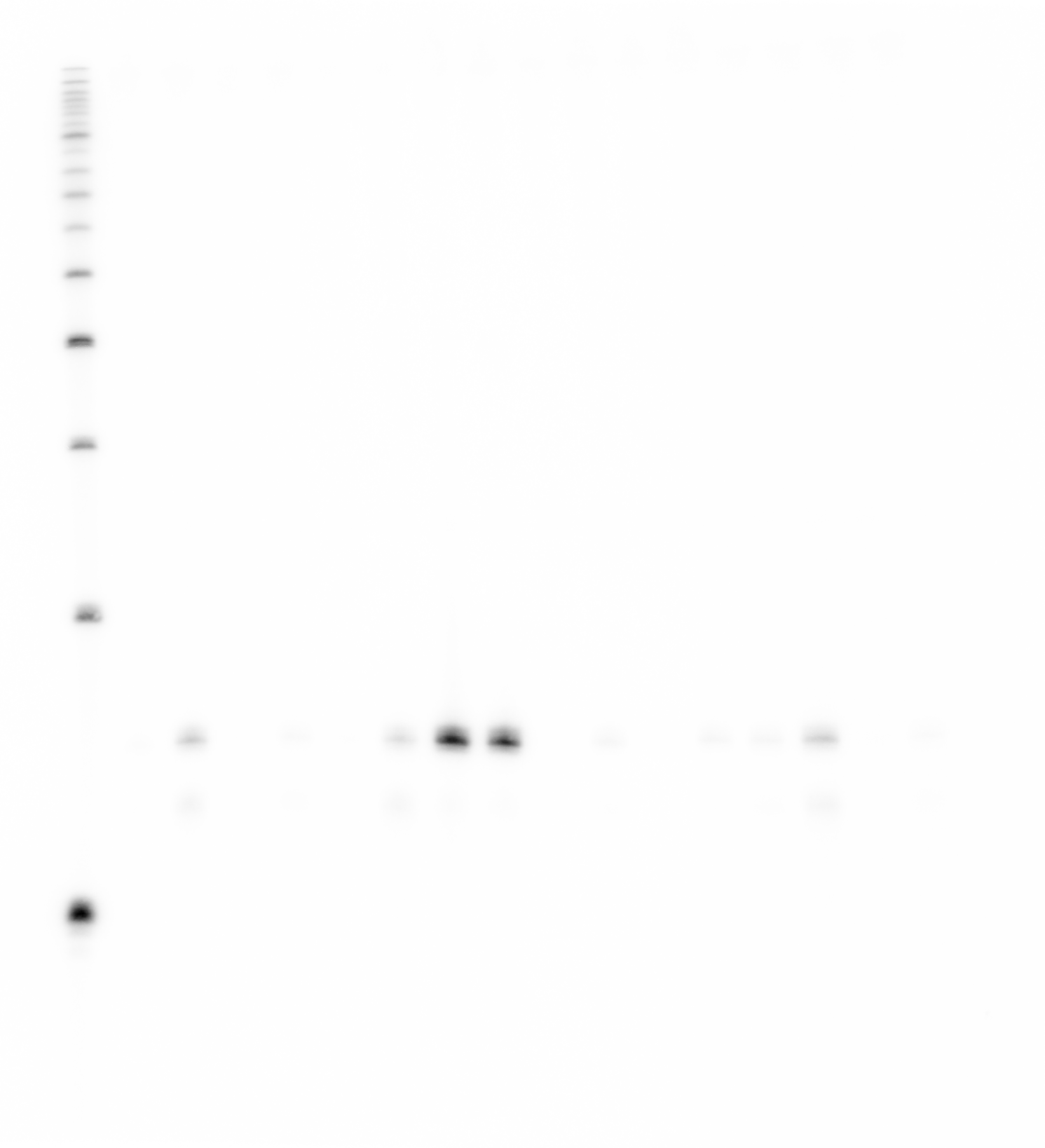

Supplement: Supplementary file 10 — Source data Fig. 1 [file 44318_2025_668_MOESM10_ESM.zip › Figure 1/1E/ChvR.tif]

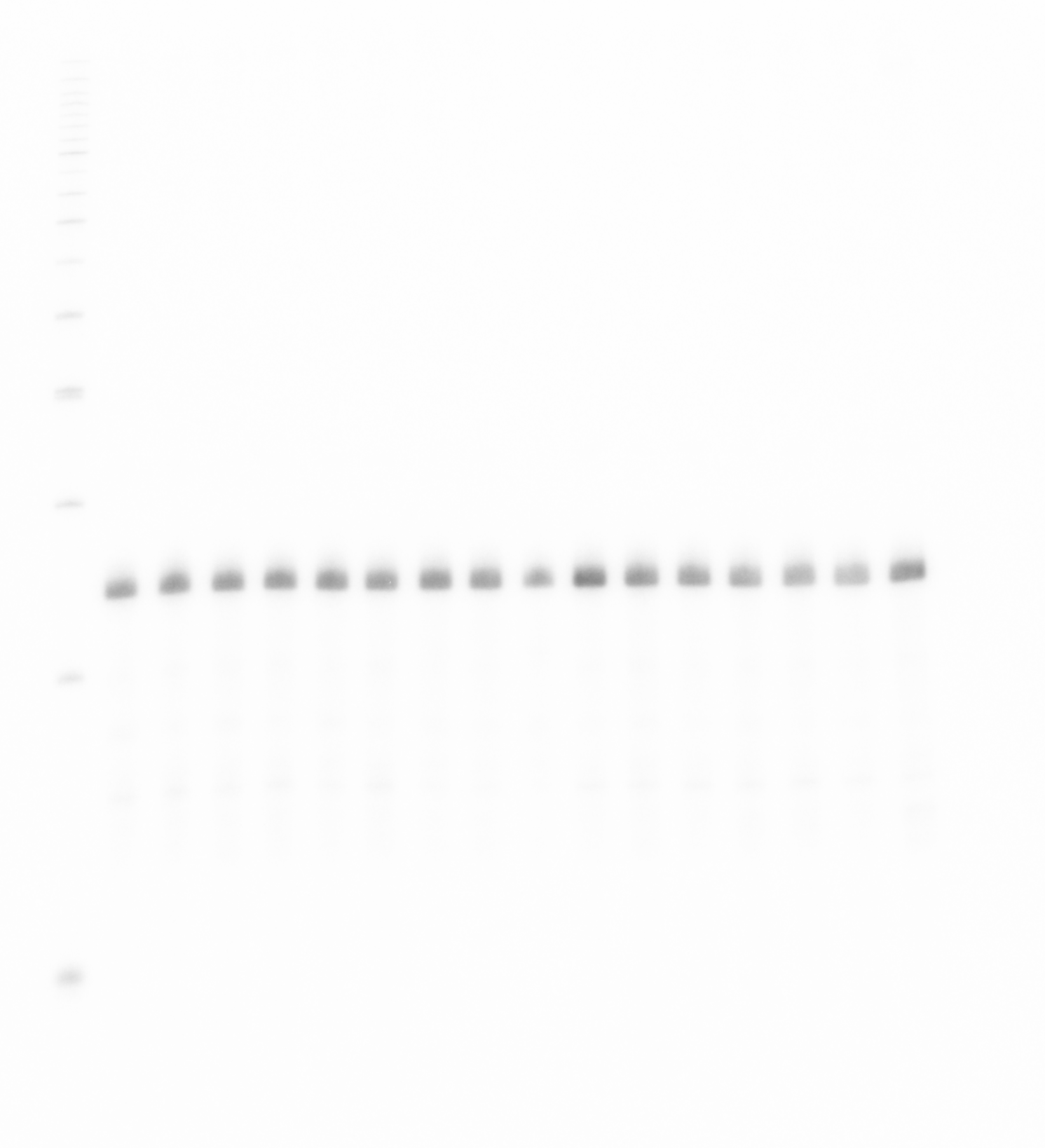

Supplement: Supplementary file 10 — Source data Fig. 1 [file 44318_2025_668_MOESM10_ESM.zip › Figure 1/1E/5S.tif]

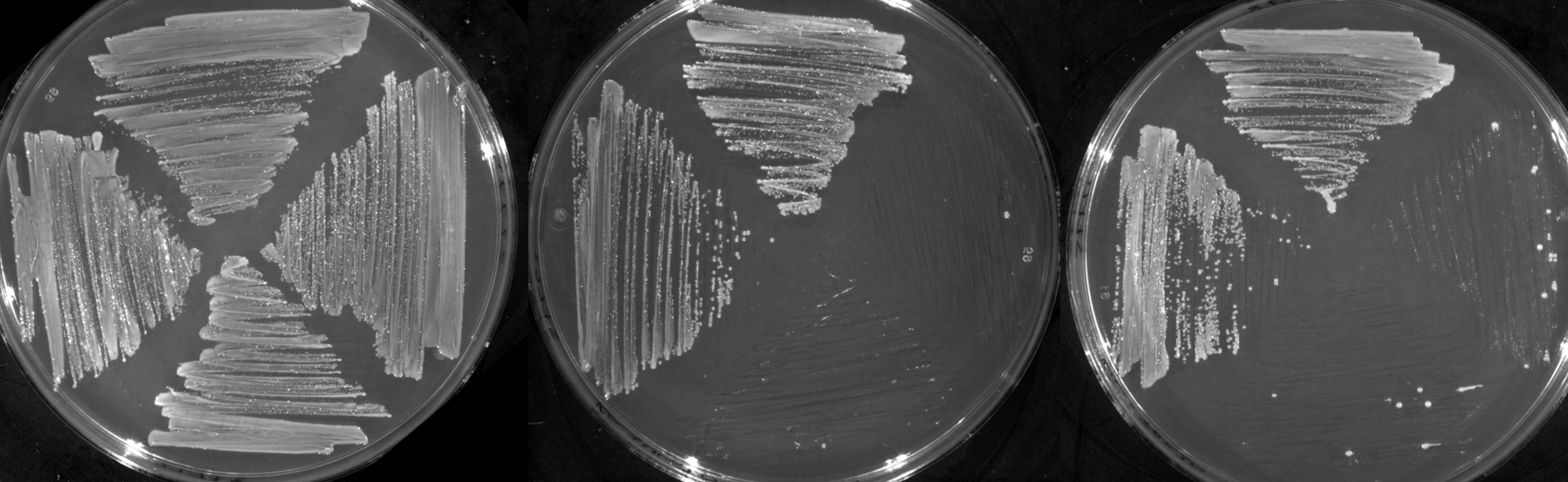

Supplement: Supplementary file 10 — Source data Fig. 1 [file 44318_2025_668_MOESM10_ESM.zip › Figure 1/1D/1D.tif]

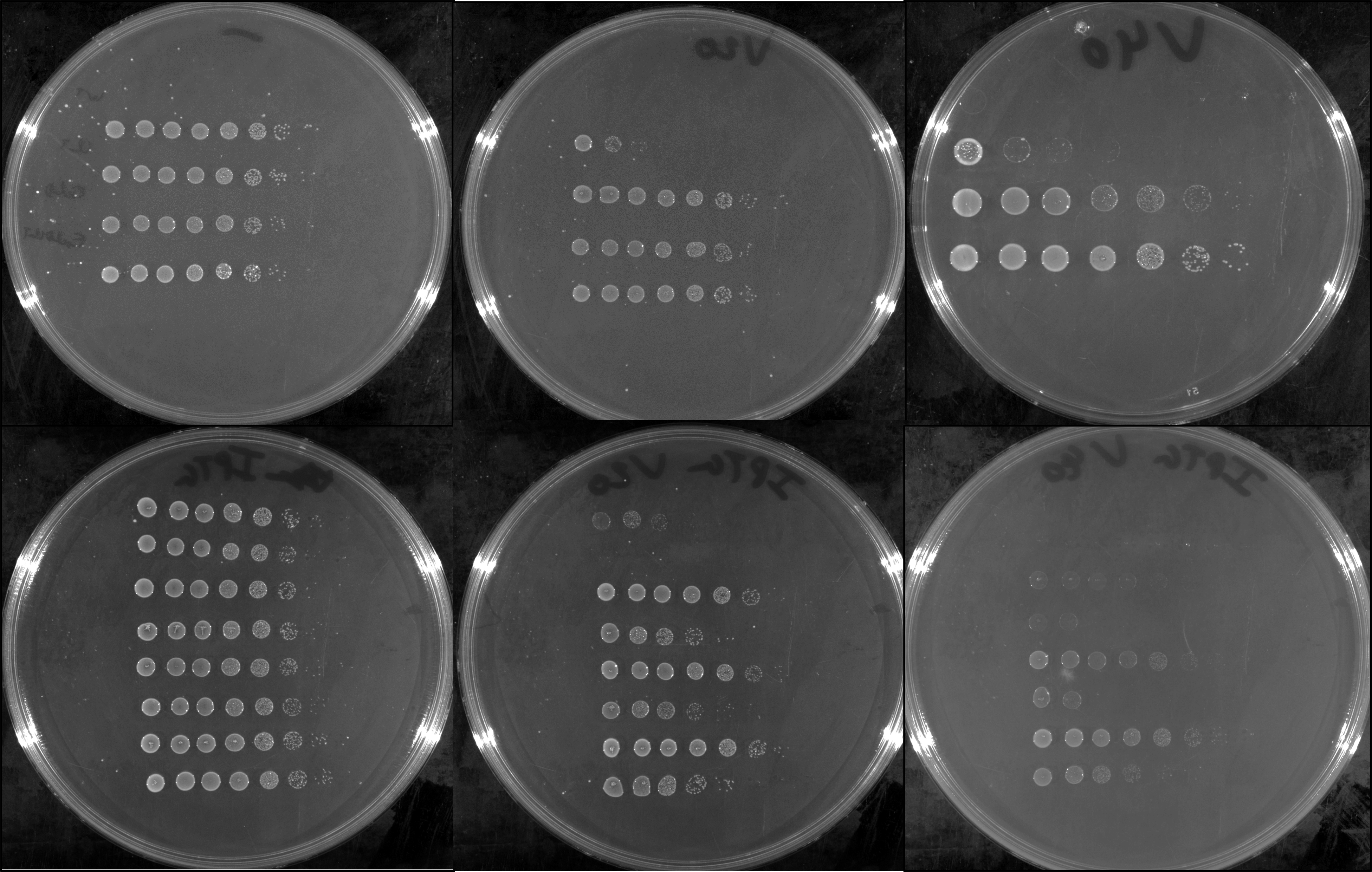

Supplement: Supplementary file 10 — Source data Fig. 1 [file 44318_2025_668_MOESM10_ESM.zip › Figure 1/1C/1C.png]

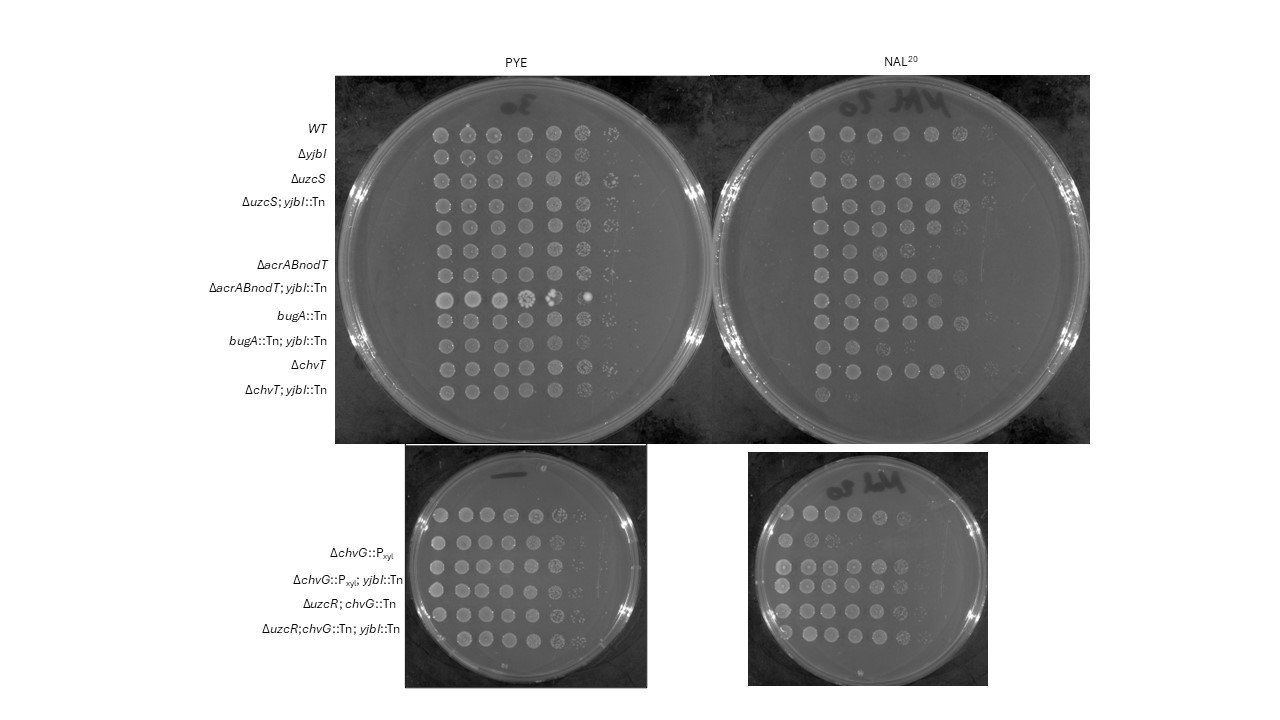

Supplement: Supplementary file 11 — Source data Fig. 2 [file 44318_2025_668_MOESM11_ESM.zip › Figure 2/2A/2A.jpg]

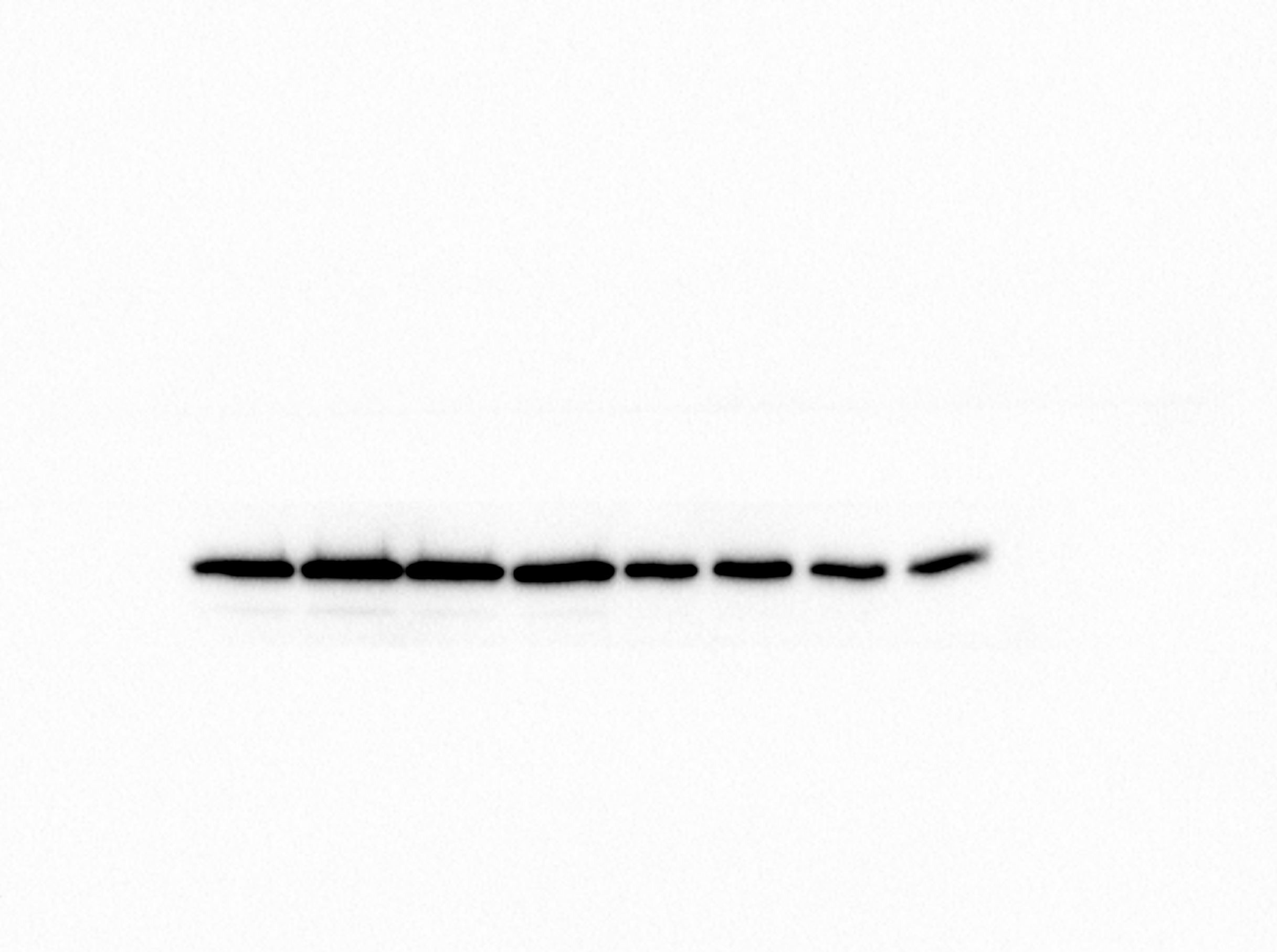

Supplement: Supplementary file 11 — Source data Fig. 2 [file 44318_2025_668_MOESM11_ESM.zip › Figure 2/2D/Control.tif]

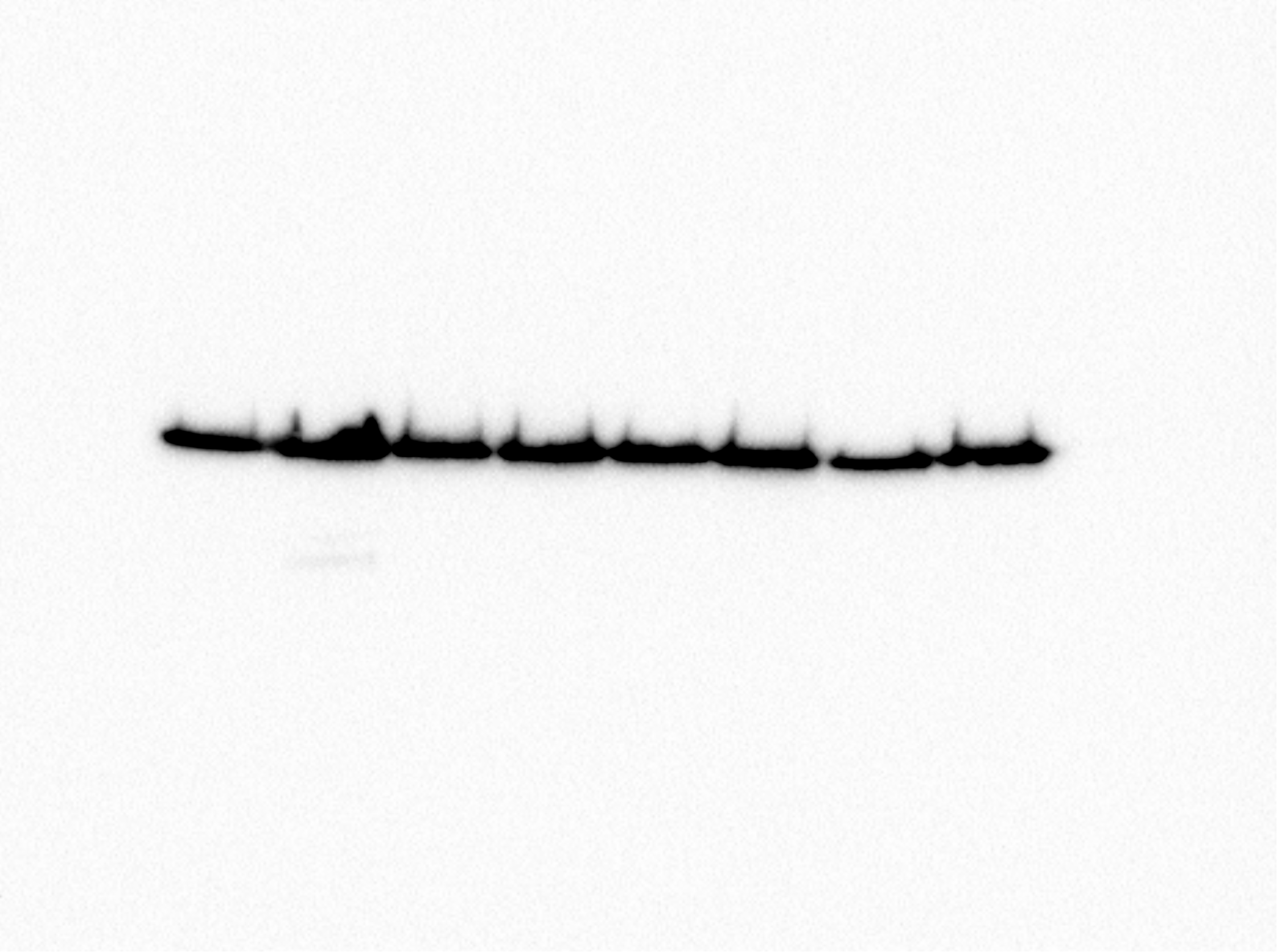

Supplement: Supplementary file 11 — Source data Fig. 2 [file 44318_2025_668_MOESM11_ESM.zip › Figure 2/2D/2D.tif]

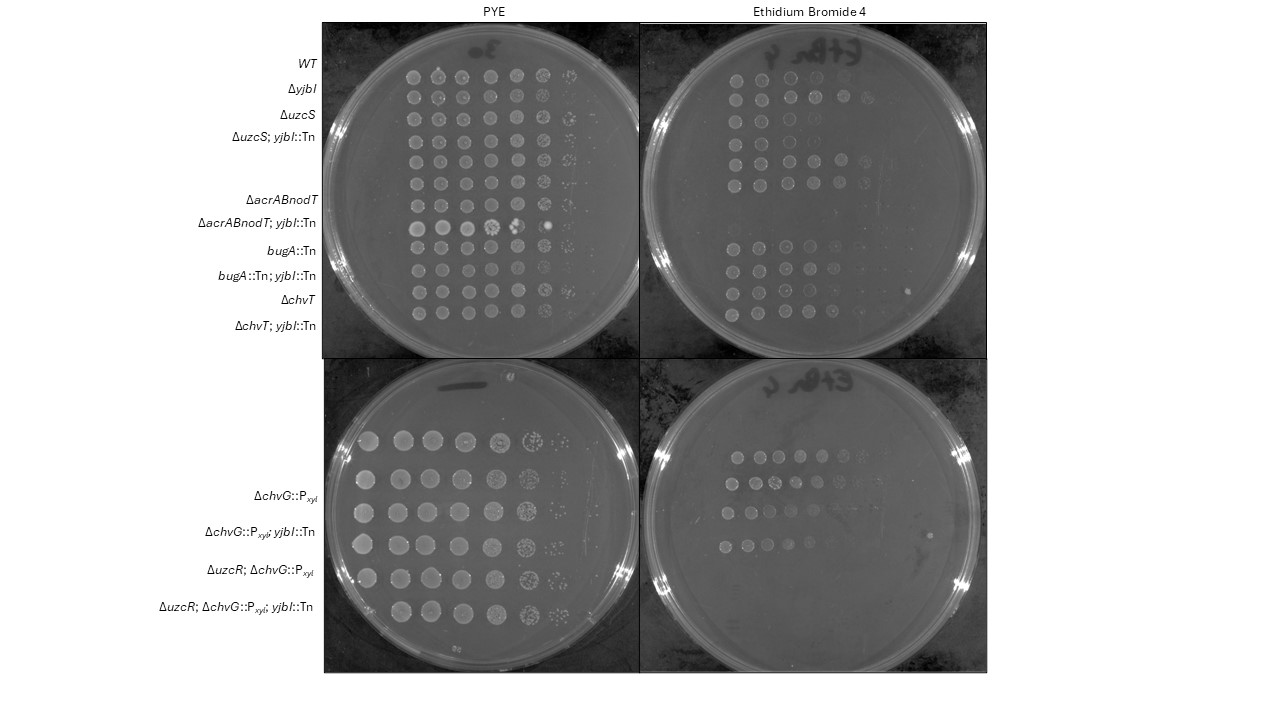

Supplement: Supplementary file 11 — Source data Fig. 2 [file 44318_2025_668_MOESM11_ESM.zip › Figure 2/2E/2E.jpg]

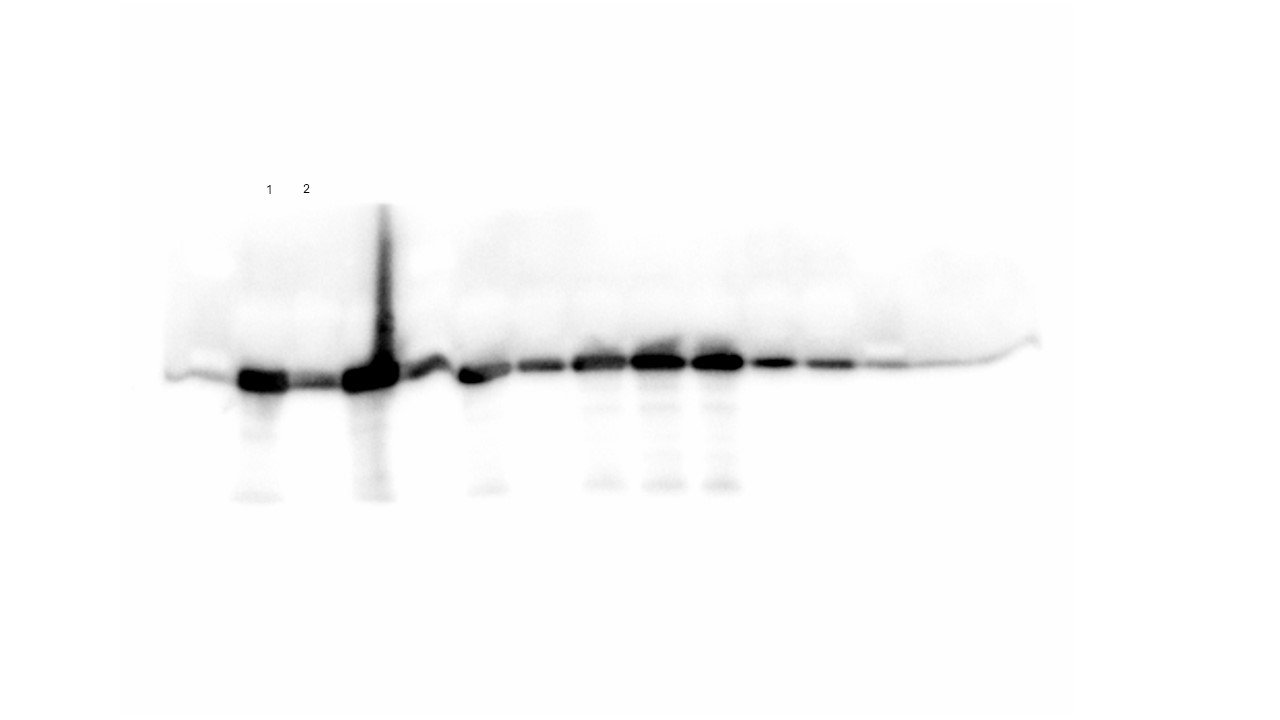

Supplement: Supplementary file 11 — Source data Fig. 2 [file 44318_2025_668_MOESM11_ESM.zip › Figure 2/2B/2B anti AcrA.jpg]

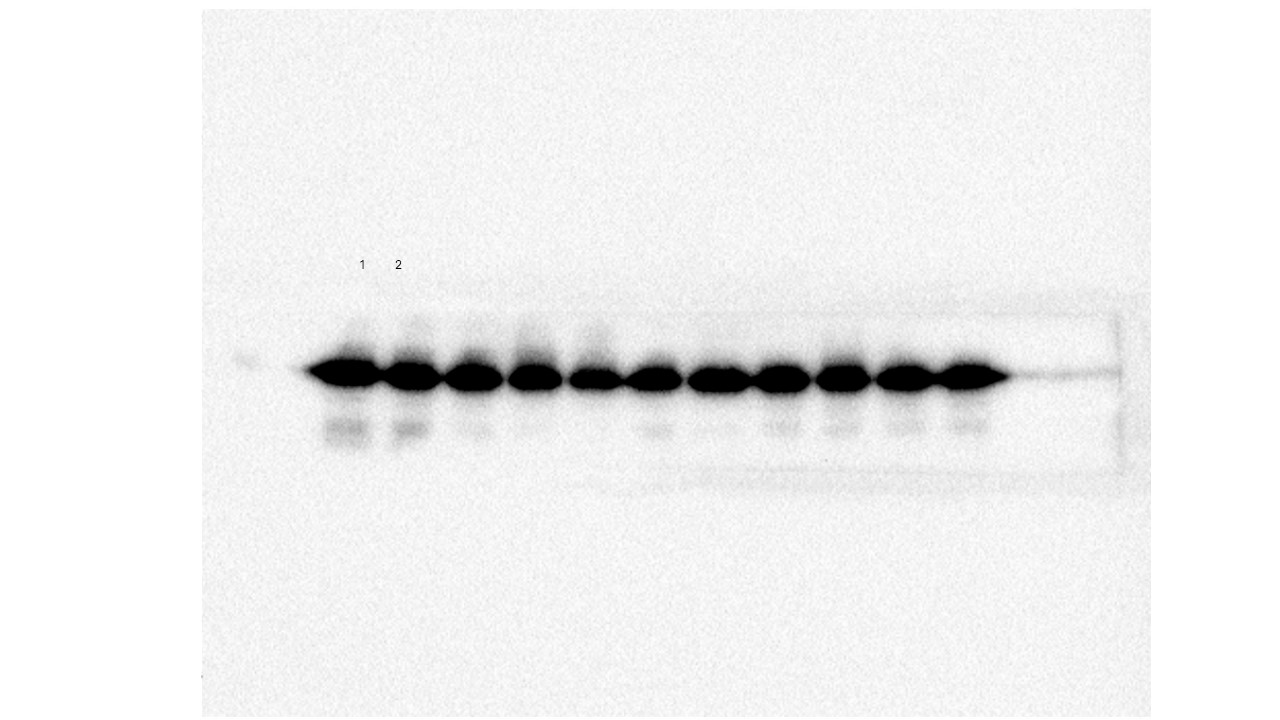

Supplement: Supplementary file 11 — Source data Fig. 2 [file 44318_2025_668_MOESM11_ESM.zip › Figure 2/2B/2B anti CCNA_00163.jpg]

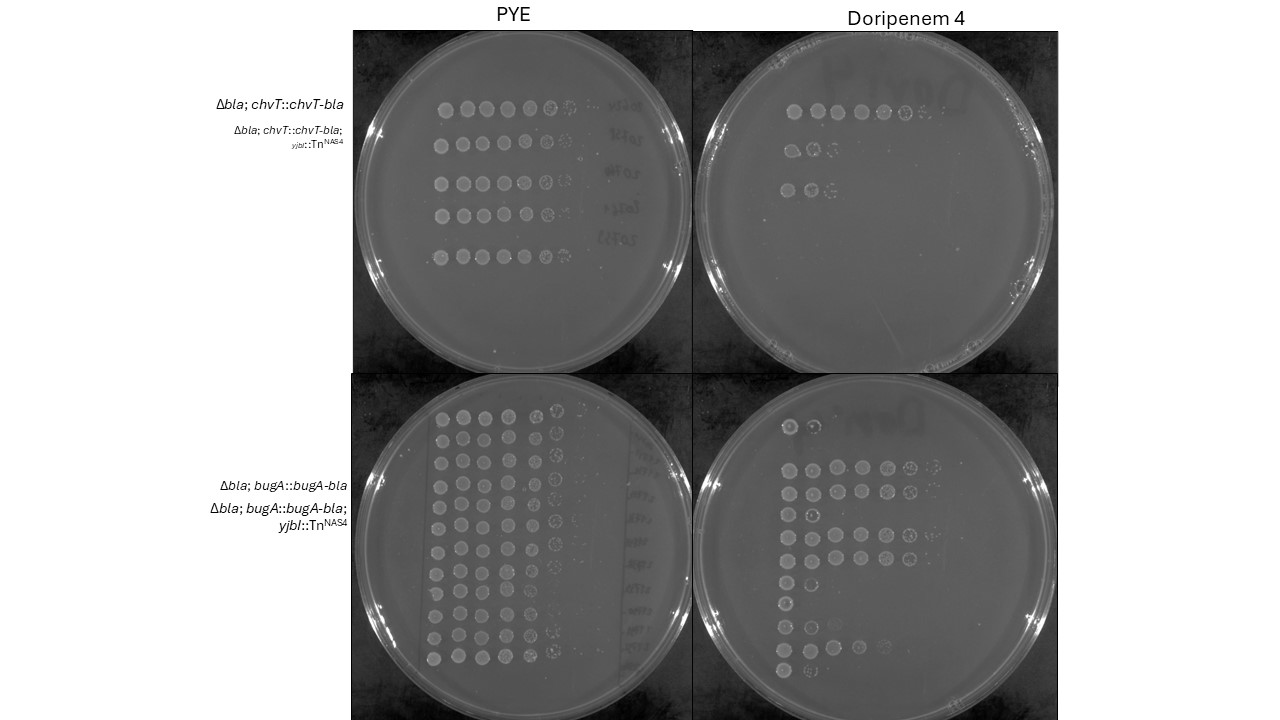

Supplement: Supplementary file 13 — Source data Fig. 4 [file 44318_2025_668_MOESM13_ESM.zip › Figure 4/4B/4B.jpg]

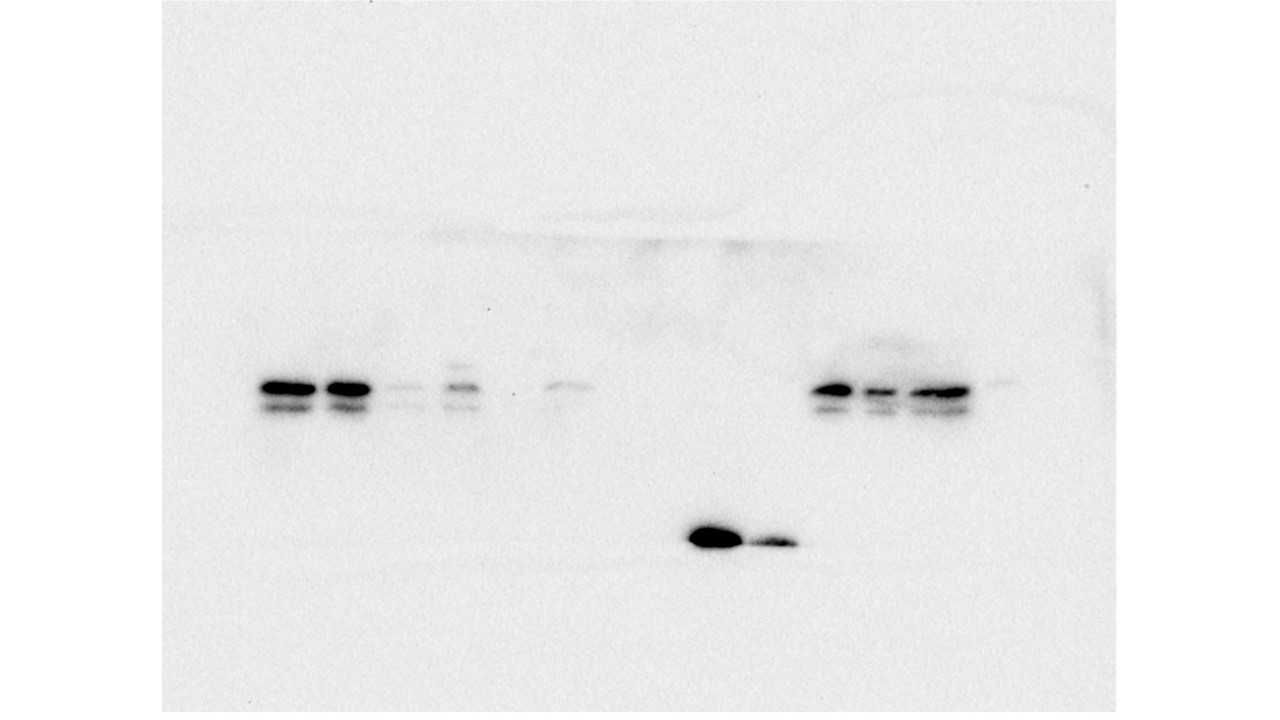

Supplement: Supplementary file 13 — Source data Fig. 4 [file 44318_2025_668_MOESM13_ESM.zip › Figure 4/4A/4A anti HA.jpg]

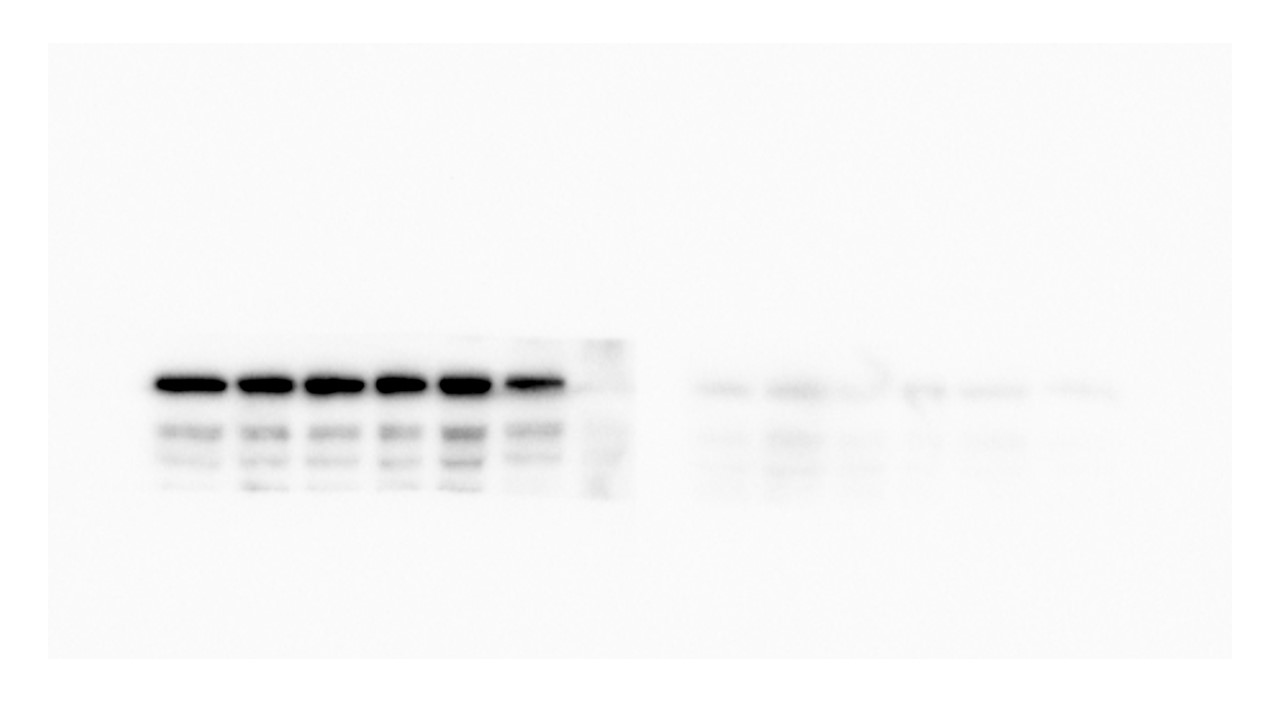

Supplement: Supplementary file 13 — Source data Fig. 4 [file 44318_2025_668_MOESM13_ESM.zip › Figure 4/4A/4A anti CCNA_00163.jpg]

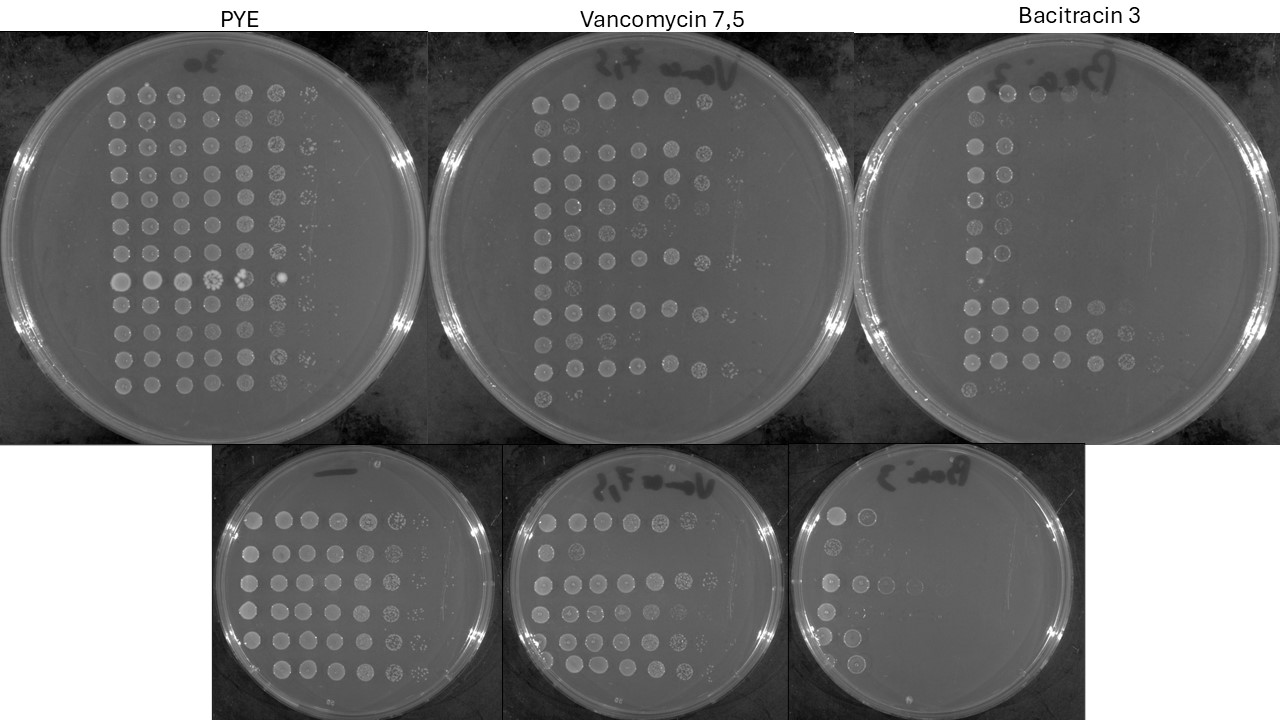

Supplement: Supplementary file 14 — Source data Fig. 5 [file 44318_2025_668_MOESM14_ESM.zip › Figure 5/5A/5A.jpg]

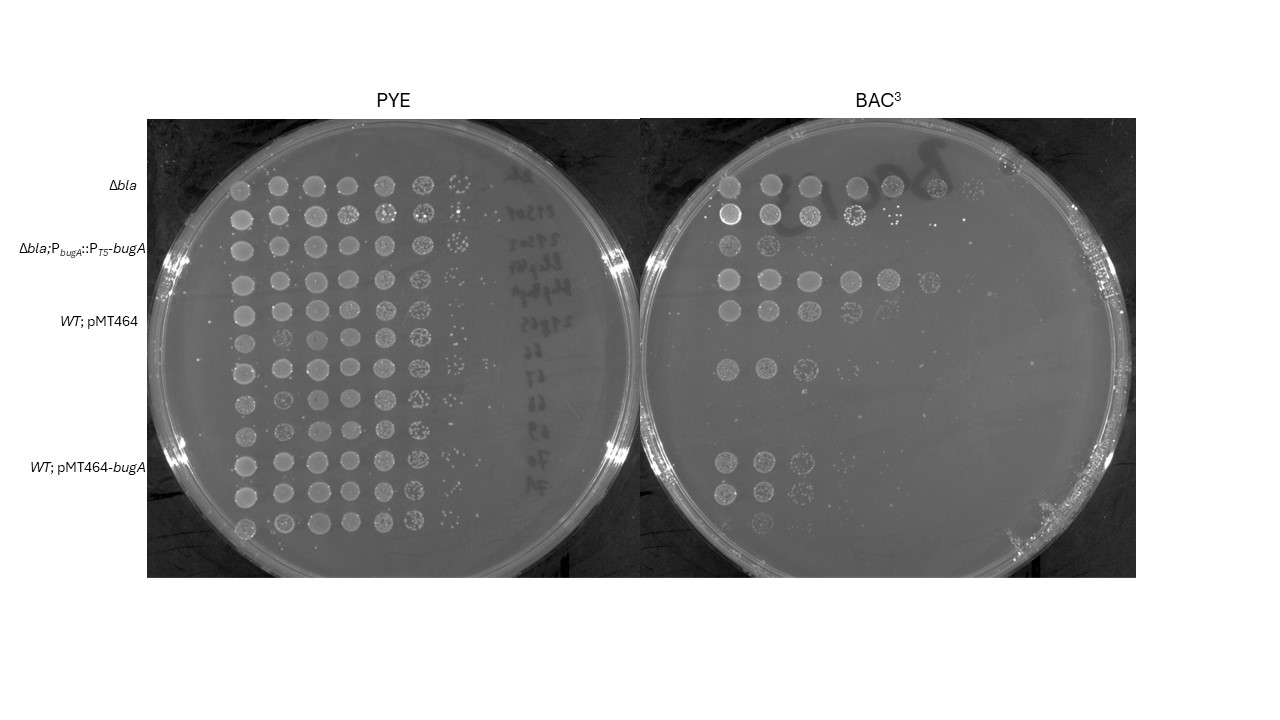

Supplement: Supplementary file 14 — Source data Fig. 5 [file 44318_2025_668_MOESM14_ESM.zip › Figure 5/5E/5E.jpg]

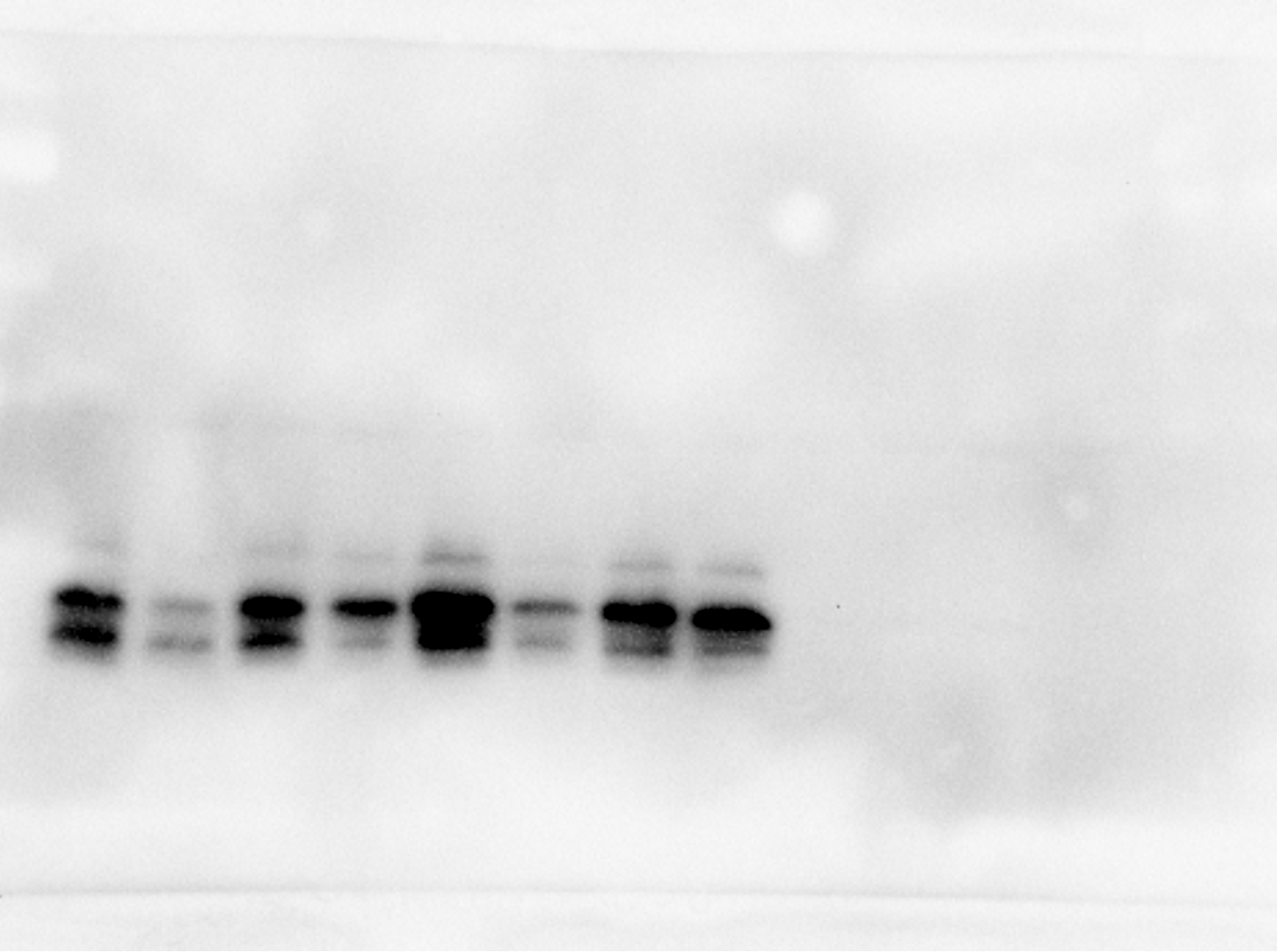

Supplement: Supplementary file 15 — Source data Fig. 6 [file 44318_2025_668_MOESM15_ESM.zip › Figure 6/6E/6E.tif]

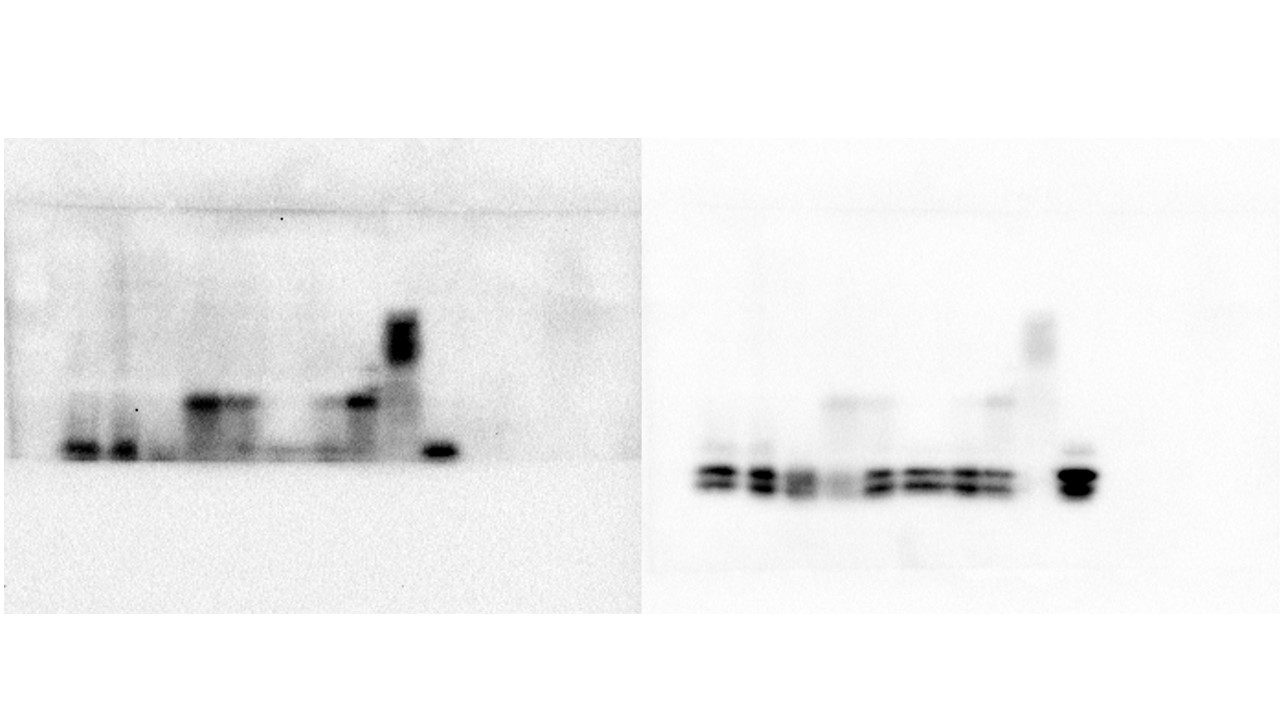

Supplement: Supplementary file 15 — Source data Fig. 6 [file 44318_2025_668_MOESM15_ESM.zip › Figure 6/6E/6E maleimide.jpg]
