# Supplementary material for: Asymmetric envelope surface disposition of secreted protein YjbI controls bimodal antibiotic susceptibilities in C. crescentus
Source: EMBO J. 2026 Jan 3;45(3):987–1023. doi: 10.1038/s44318-025-00668-x (PMC12864828; doi:10.1038/s44318-025-00668-x)
Supplement: Supplementary file 16 — Expanded View Figures [file 44318_2025_668_MOESM16_ESM.pdf]

Expanded View Figures

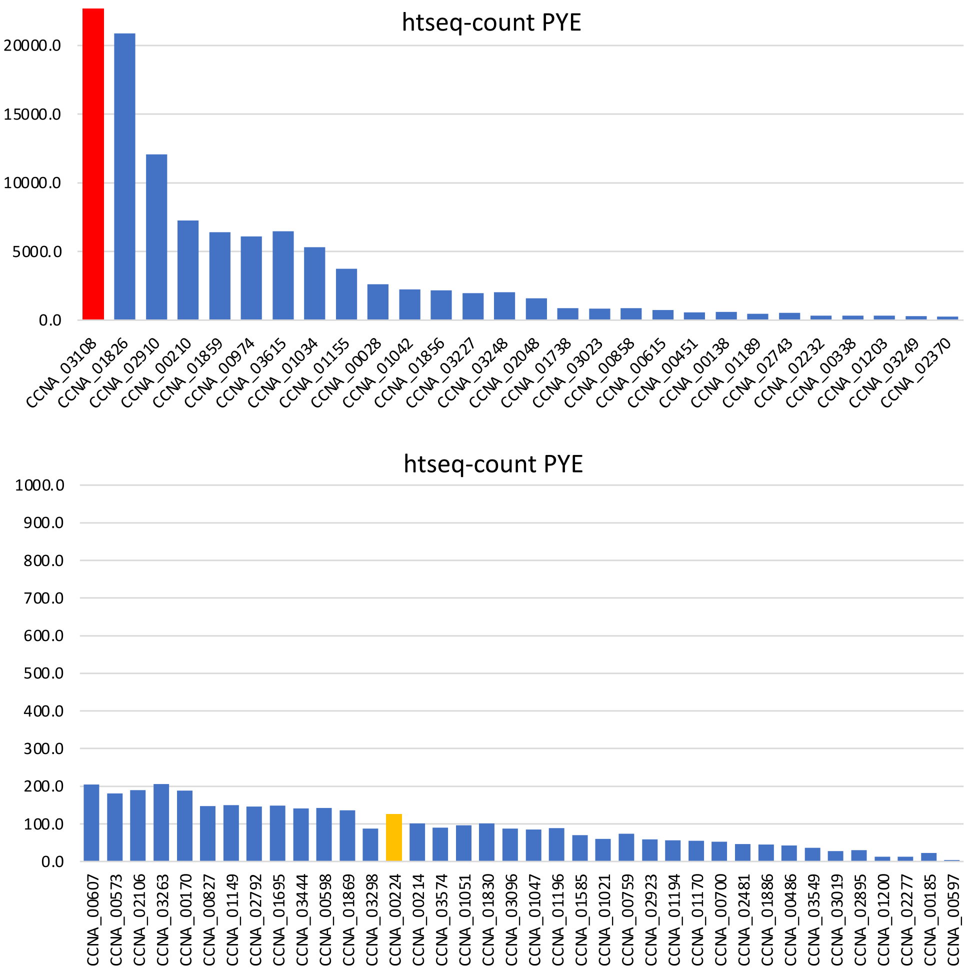

Figure EV1. Expression of TBDRs as determined by RNA-Seq.

Graphical representation of the htseq transcript count of RNA-Seq data from (Siwach et al, 2021) showing the expression profiles of all annotated TBDR genes from *Caulobcater crescentus* NA1000 grown in PYE. Note that the *chvT* transcript (*CCNA_03108*, in red) and the *bugA* transcript (*CCNA_00224* in yellow) are at opposite ends of the abundance spectrum of TBDR-encoding transcripts, with *chvT* being the most abundant, whilst the *bugA* transcript is among the low abundance transcripts.

© The Author(s)

A

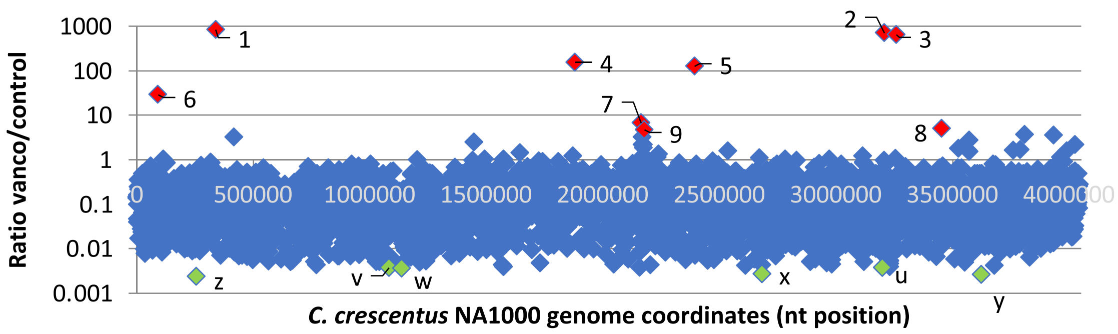

B

#	ID (feature)	Description	Ratio
1	CCNA_00324 (exbD)	ExbD-family protein	832.31
2	CCNA_03052	acyltransferase	702.05
3	CCNA_03108 (chvT)	TonB-dependent outer membrane receptor	643.50
4	CCNA_01751 (mltG)	endolytic murein transglycosylase	156.10
5	CCNA_02243 (crbA)	D-alanyl-D-alanine serine-type carboxypeptidase	125.41
6	CCNA_00086 (gdhZ)	NAD-specific glutamate dehydrogenase	28.71
7	CCNA_02016 (nuoM)	NADH-quinone oxidoreductase chain M	6.64
8	CCNA_03281	Lrp-family transcriptional regulator	5.03
9	CCNA_02033 (nuoA)	NADH-quinone oxidoreductase chain A	4.70
u	CCNA_03043 (pilA)	type IV pilin protein	0.00377
v	CCNA_00998 (mucR2)	ROS/MUCR transcriptional regulator	0.00372
w	CCNA_01041	hypothetical protein	0.00367
x	CCNA_02533	hypothetical protein	0.00266
y	CCNA_03992	UDP-glucose 6-dehydrogenase-related protein	0.00261
z	CCNA_03917	hypothetical protein	0.00235

C

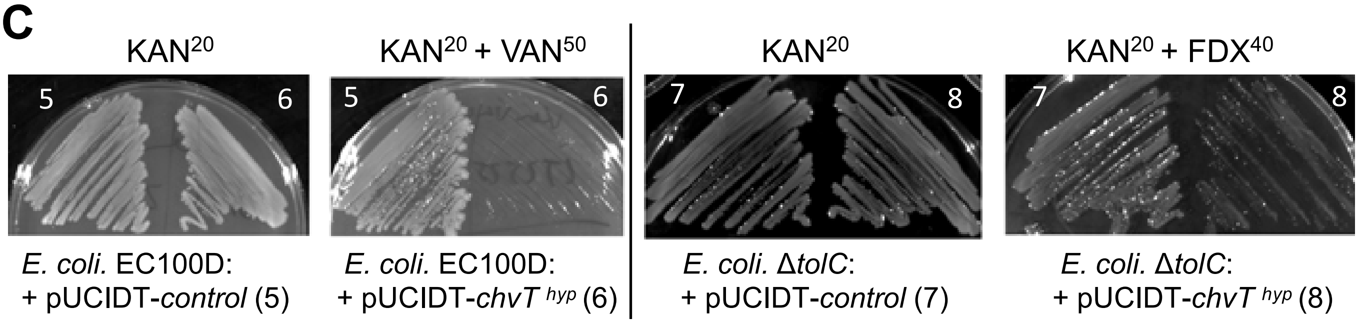

D

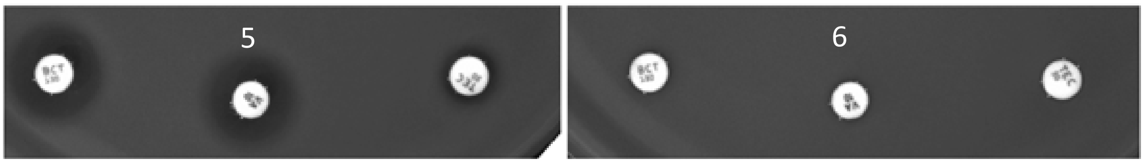

© The Author(s)

◀ **Figure EV2. Vancomycin resistance determinants identified by Tn-Seq.**

(**A**) Plot representation of the Tn-Seq on PYE plates containing 20 μg/mL of vancomycin (VAN^{20}). (**B**) Table showing the top nine genes ("1–9") whose disruption favors growth on plates with VAN^{20} and are thus overrepresented as Tn insertion sites, while the six lowest (underrepresented genes) are also shown in the table indicated as positions "u-z". (**C**) Growth of *E. coli* EC100D or efflux-defective Δ*tolC* cells containing pUCIDT- control or pUCIDT-*chvT* ^{hyp} plasmids streaked on LB-plates containing 20 μg/mL of kanamycin (KAN^{20}) with or without or 50 μg/mL of vancomycin (VAN^{50}) or 40 μg/mL fidaxomicin (FDX^{40}). Since FDX is a substrate of the *E. coli* AcrAB-TolC multidrug efflux pump, the permeability of FDX conferred by $ChvT^{hyp}$ expression is best detectable in Δ*tolC* cells. (**D**) Growth inhibition assays by antibiotics on discs placed on *E. coli* EC100D cells embedded on LB-soft agar on LB plates. Discs from left to right containing 130 units bacitracin (BCT130), 10 μg/mL of vancomycin (VA10) or 30 μg/mL of teicoplanin (TEC30). Plates were incubated for 2 days at 30 °C.

© The Author(s)

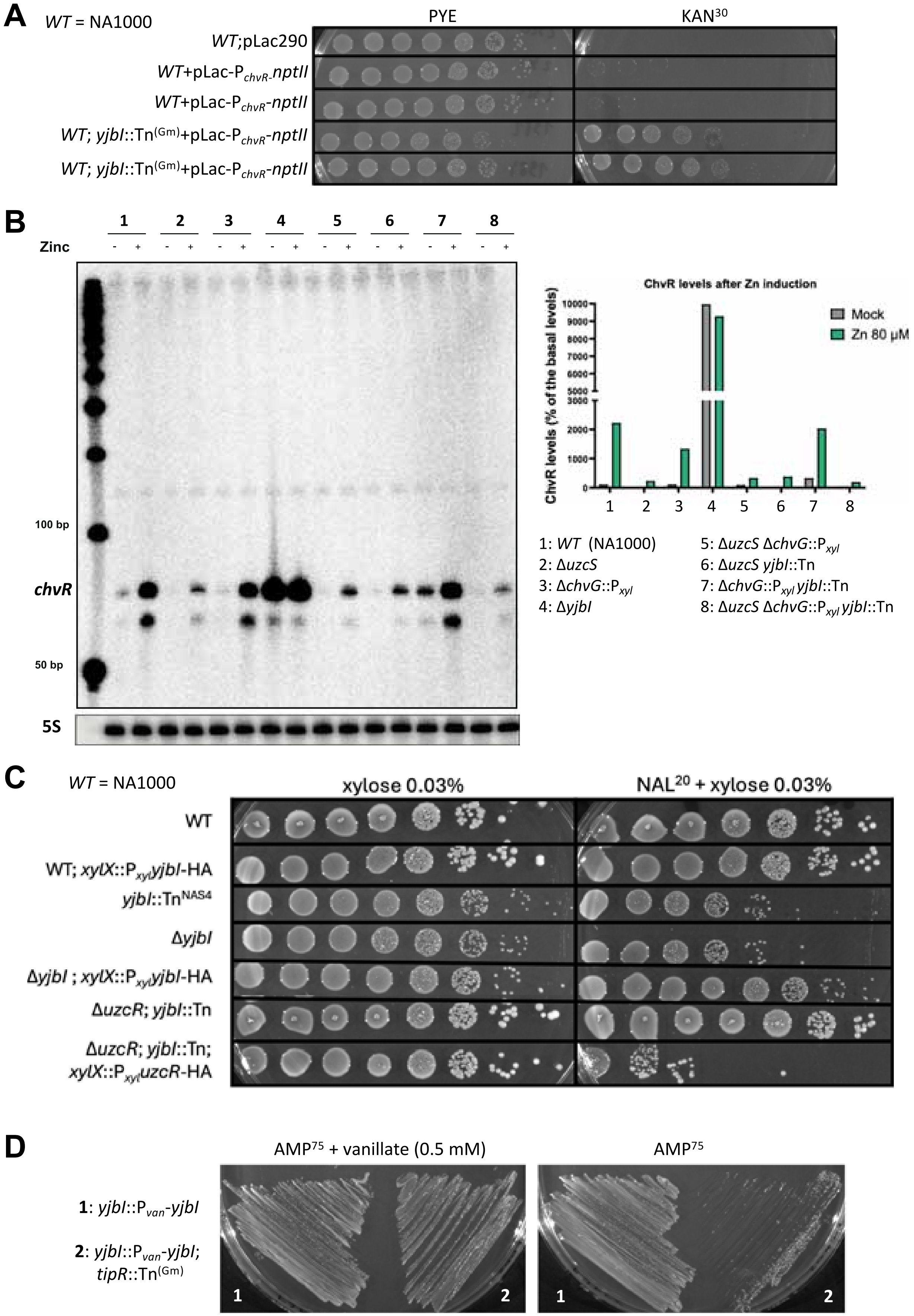
A
WT = NA1000
PYE
KAN30
WT;pLac290
WT+pLac-PchvR-nptII
WT+pLac-PchvR-nptII
WT; yjbI::Tn(Gm)+pLac-PchvR-nptII
WT; yjbI::Tn(Gm)+pLac-PchvR-nptII
B
1 2 3 4 5 6 7 8
Zinc
- + - + - + - + - + - + - + - +
100 bp
chvR
50 bp
5S
ChvR levels after Zn induction
ChvR levels (% of the basal levels)
Mock
Zn 80 µM
1: WT (NA1000)
2: ΔuzcS
3: ΔchvG::Pxyl
4: ΔyjbI
5: ΔuzcS ΔchvG::Pxyl
6: ΔuzcS yjbI::Tn
7: ΔchvG::Pxyl yjbI::Tn
8: ΔuzcS ΔchvG::Pxyl yjbI::Tn
C
WT = NA1000
xylose 0.03%
NAL20 + xylose 0.03%
WT
WT; xylX::PxylyjbI-HA
yjbI::TnNAS4
ΔyjbI
ΔyjbI ; xylX::PxylyjbI-HA
ΔuzcR; yjbI::Tn
ΔuzcR; yjbI::Tn;
xylX::PxyluzcR-HA
D
AMP75 + vanillate (0.5 mM)
AMP75
1: yjbI::Pvan-yjbI
2: yjbI::Pvan-yjbI;
tipR::Tn(Gm)
1
2
1
2

© The Author(s)

Figure EV3. Phenotype of cells lacking YjbI.

(**A**) EOP assay of strains carrying the pLac290 empty vector or the pLac290 derivative harboring the P_{chvR} - *nptII* reporter plated PYE with or without 30 µg/mL kanamycin (KAN^{30}). Plates were incubated for 2 days at 30 °C. (**B**) Northern blots are shown as described in the inset of Fig. 1D. This figure shows the full blot and size markers. The graph on the right is a quantification of the transcripts detected by Northern blot. (**C**) EOP assay of *yjbI* mutant cells complemented with YjbI-HA or *uzcR* mutant complemented with HA-UzcR on PYE containing or not 20 µg/mL of nalidixic acid (NAL^{20}). Both plates contain 0.03% of xylose and were incubated for 48 h at 30 °C. (**D**) Growth of *yjbI*::P_{van}-*yjbI* cells with or without *tipR*::Tn on plates containing 75 µg/mL of ampicillin (AMP75) with or without 0.5 mM vanillate. Plates were incubated for 60 h at 30 °C.

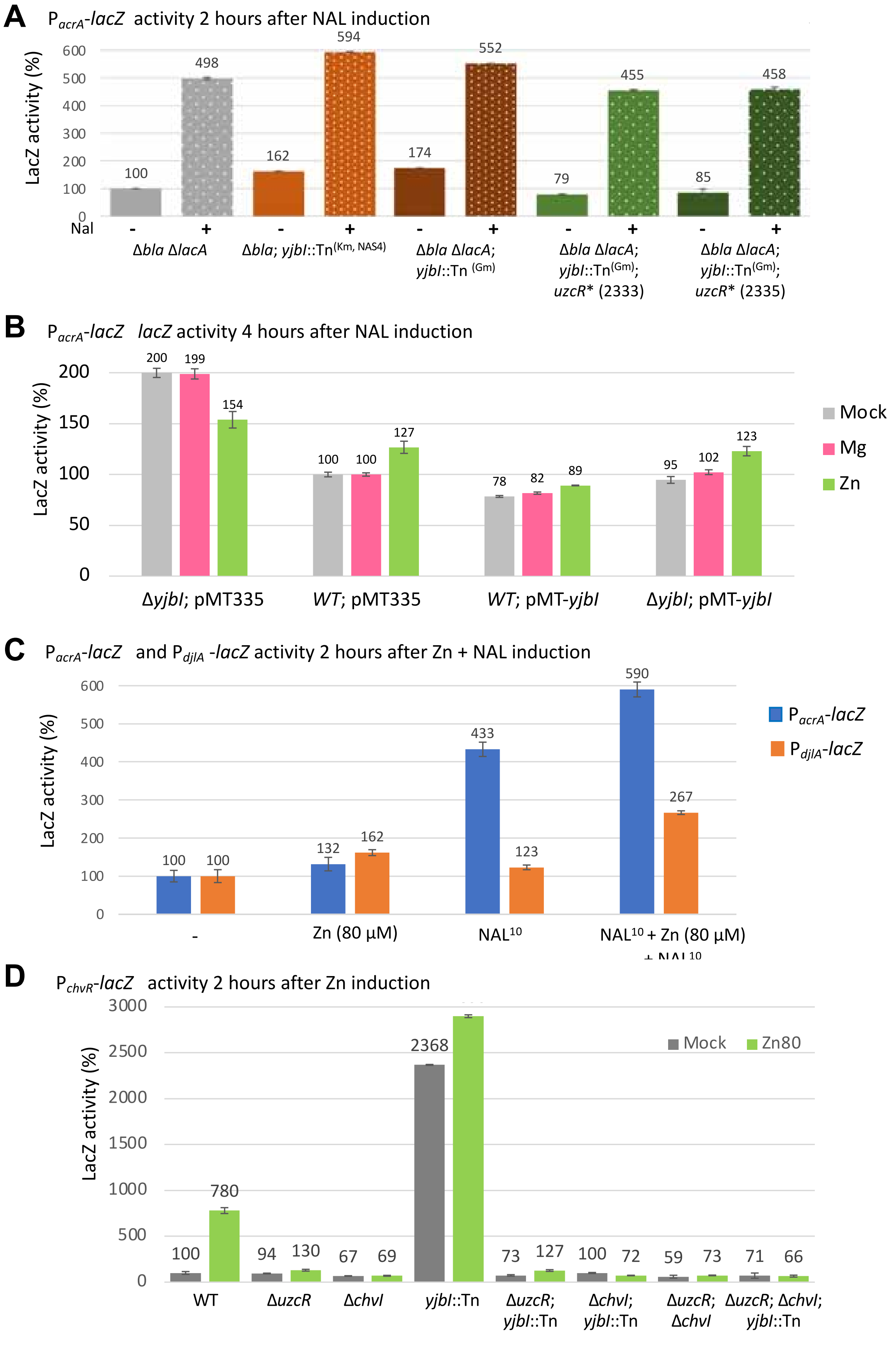
A
PacrA-lacZ activity 2 hours after NAL induction
LacZ activity (%)
100
498
162
594
174
552
79
455
85
458
Nal
Δbla ΔlacA
Δbla; yjbI::Tn(Km, NAS4)
Δbla ΔlacA; yjbI::Tn (Gm)
Δbla ΔlacA; yjbI::Tn(Gm); uzcR* (2333)
Δbla ΔlacA; yjbI::Tn(Gm); uzcR* (2335)
B
PacrA-lacZ lacZ activity 4 hours after NAL induction
LacZ activity (%)
Mock
Mg
Zn
200
199
154
100
100
127
78
82
89
95
102
123
ΔyjbI; pMT335
WT; pMT335
WT; pMT-yjbI
ΔyjbI; pMT-yjbI
C
PacrA-lacZ and PdjlA -lacZ activity 2 hours after Zn + NAL induction
LacZ activity (%)
PacrA-lacZ
PdjlA-lacZ
100
100
132
162
433
123
590
267
-
Zn (80 µM)
NAL10
NAL10 + Zn (80 µM)
D
PchvR-lacZ activity 2 hours after Zn induction
LacZ activity (%)
Mock
Zn80
100
780
94
130
67
69
2368
73
127
100
72
59
73
71
66
WT
ΔuzcR
ΔchvI
yjbI::Tn
ΔuzcR; yjbI::Tn
ΔchvI; yjbI::Tn
ΔuzcR; ΔchvI
ΔuzcR; ΔchvI; yjbI::Tn

© The Author(s)

Figure EV4. NAL- and Zn induction in cells lacking YjbI.

(**A–D**) β-galactosidase assay using the pP_{acrA}-*lacZ* (**A–C**), pP_{djlA}-*lacZ* (**C**) and pP_{chvR}-*lacZ* (**D**) promoter probe plasmids in various mutants performed for two (**A**, **B**, **D**) or 4 h (**C**) with $MgSO_4$, $ZnSO_4$ (80 µM) or NAL[10] (+) at 30 °C in PYE. All levels are indicated in percentage of expression relative to the basal level of the *WT* in the uninduced condition. Error bars are defined as +/− standard deviation. All experiments done with strains carrying the pMT335 (and derivatives) were done in the presence of vanillate (100 µM).

© The Author(s)

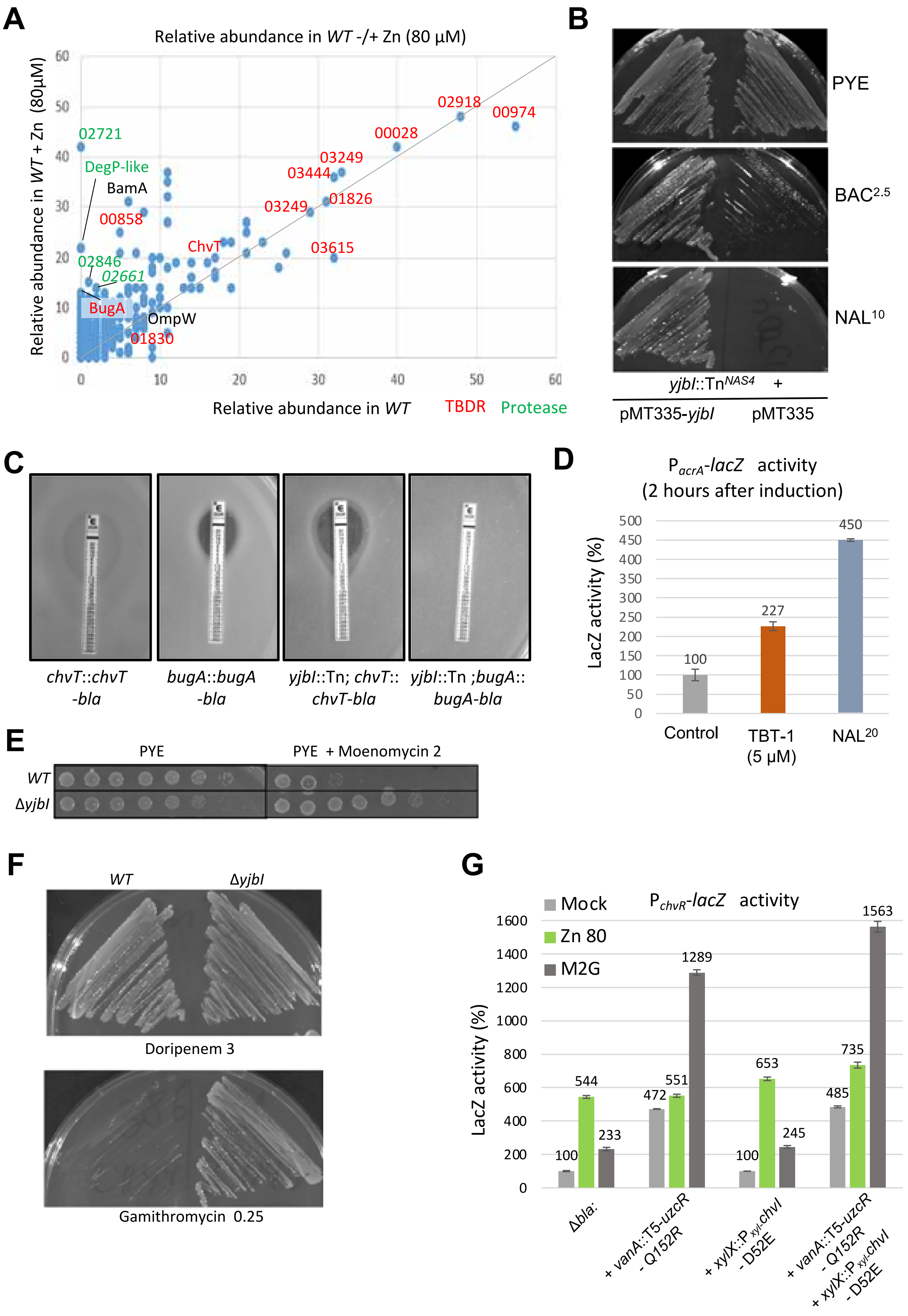
A
Relative abundance in WT -/+ Zn (80 µM)
Relative abundance in WT + Zn (80µM)
Relative abundance in WT
TBDR
Protease
02721
DegP-like
BamA
00858
02846
02661
BugA
OmpW
01830
ChvT
03249
03444
01826
03615
00028
02918
00974
B
PYE
BAC2.5
NAL10
yjbI::TnNAS4 +
pMT335-yjbI
pMT335
C
chvT::chvT -bla
bugA::bugA -bla
yjbI::Tn; chvT:: chvT-bla
yjbI::Tn ;bugA:: bugA-bla
D
PacrA-lacZ activity
(2 hours after induction)
LacZ activity (%)
100
227
450
Control
TBT-1 (5 µM)
NAL20
E
PYE
PYE + Moenomycin 2
WT
ΔyjbI
F
WT
ΔyjbI
Doripenem 3
Gamithromycin 0.25
G
PchvR-lacZ activity
Mock
Zn 80
M2G
LacZ activity (%)
100
544
233
472
551
1289
100
653
245
485
735
1563
Δbla:
+ vanA::T5-uzcR - Q152R
+ xylX::Pxyl-chvI - D52E
+ vanA::T5-uzcR - Q152R + xylX::Pxyl-chvI - D52E

 © The Author(s)

Figure EV5. OM proteome, ChvT and BugA expression in *WT* and *yjbI* mutant cells.

(**A**) Scatter plot graphic representing the relative abundancy of proteins from outer membrane enriched samples of WT cells after 2 h of growth with or without $ZnSO_4$ (80 µM). The TBDR are indicated in red while the predicted proteases are in green. (**B**) Complementation tests of *yjbI*::Tn^{NAS4} mutants harboring pMT335 or pMT335-*yjbI* streaked on PYE plates, plates with NAL^{10} or plates with BAC$^{2.5}$. (**C**) Doripenem MIC estimation by E-test, a strip containing various concentrations of doripenem. The E-test was placed on soft agar seeded with Δ*bla chvT*::*chvT-bla* and Δ*bla bugA*::*bugA-bla* cells with or without the *yjbI*::Tn^{NAS4} mutation. The plates were incubated for 24 h at 30 °C. (**D**) β-galactosidase assay using the pP$_{acrA}$-*lacZ* promoter probe plasmid in *WT* cells following 2 h of induction with TBT-1 or with NAL at 30 °C in PYE. Error bars are defined as +/- standard deviation. (**E**) EOP assay of *WT* and Δ*yjbI* cells on PYE plates with or without moenomycin (2 µg/mL). The plates were incubated for 2 days at 30 °C. (**F**) *WT* and Δ*yjbI* cells were streaked on agar plates containing either doripenem (3 µg/mL) or gamithromycin (0.25 µg/mL) and grown for 2 days at 30 °C. (**G**) β-galactosidase assay using the pP$_{chvR}$-*lacZ* promoter probe plasmid in cells expressing UzR-Q152R from the strong *E. coli* T5 promoter at the *vanA* locus or ChvI-D52E from the P$_{xyl}$ promoter at the *xylX* locus. Cells were grown in PYE containing 0.3% xylose, exposed to $ZnSO_4$ (80 µM) for 2 h or, switched and grown in M2G minimal medium for 5 h before measurements. Error bars are defined as +/− standard deviation.

© The Author(s)

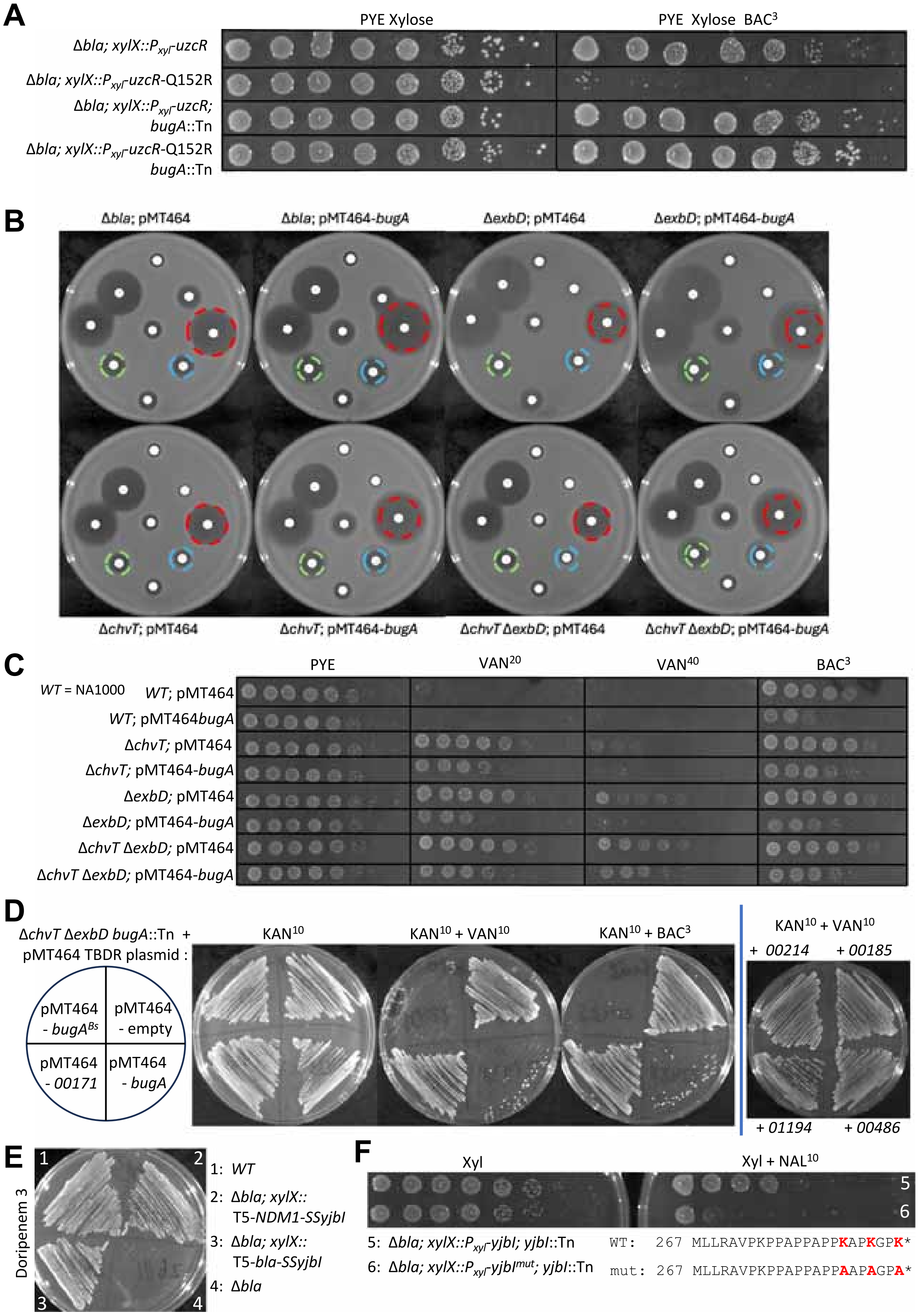
A
PYE Xylose
PYE Xylose BAC3
Δbla; xylX::Pxyl-uzcR
Δbla; xylX::Pxyl-uzcR-Q152R
Δbla; xylX::Pxyl-uzcR; bugA::Tn
Δbla; xylX::Pxyl-uzcR-Q152R bugA::Tn
B
Δbla; pMT464
Δbla; pMT464-bugA
ΔexbD; pMT464
ΔexbD; pMT464-bugA
ΔchvT; pMT464
ΔchvT; pMT464-bugA
ΔchvT ΔexbD; pMT464
ΔchvT ΔexbD; pMT464-bugA
C
PYE
VAN20
VAN40
BAC3
WT = NA1000
WT; pMT464
WT; pMT464bugA
ΔchvT; pMT464
ΔchvT; pMT464-bugA
ΔexbD; pMT464
ΔexbD; pMT464-bugA
ΔchvT ΔexbD; pMT464
ΔchvT ΔexbD; pMT464-bugA
D
ΔchvT ΔexbD bugA::Tn + pMT464 TBDR plasmid :
pMT464 - bugABs
pMT464 - empty
pMT464 - 00171
pMT464 - bugA
KAN10
KAN10 + VAN10
KAN10 + BAC3
KAN10 + VAN10
+ 00214
+ 00185
+ 01194
+ 00486
E
Doripenem 3
1
2
3
4
1: WT
2: Δbla; xylX:: T5-NDM1-SSyjbI
3: Δbla; xylX:: T5-bla-SSyjbI
4: Δbla
F
Xyl
Xyl + NAL10
5
6
5: Δbla; xylX::Pxyl-yjbI; yjbI::Tn
6: Δbla; xylX::Pxyl-yjbImut; yjbI::Tn
WT: 267 MLLRAVPKPPAPPAPPKAPKGPK*
mut: 267 MLLRAVPKPPAPPAPPAAPAGPA*

Figure EV6. Antibiotic sensitivity analysis of cells expressing TBDRs.

(**A**) EOP assay of Δ*bla* cells harboring *xyl*::P_{xyl}-*uzcR* or *xyl*::P_{xyl}-*uzcR-Q152R* with or without the *bugA*::Tn mutation. Cells were plated on PYE plates containing 0.3% xylose, with or without BAC3 and the plates were incubated for 2 days at 30 °C. (**B**) Kirby-Bauer type antibiograms of various *C. crescentus* strains containing pMT464-*bugA* or the empty vector. The indicator strains were embedded in PYE soft agar and antibiotic discs were placed on soft agar from top left to bottom right: colistin (50 µg), fosfomycin (50 µg), piperacillin (100 µg), rifampicin (30 µg), moenomycin (5 µg), bacitracin (130 µg), teicoplanin (30 µg), vancomycin (30 µg) and ramoplanin (40 µg). Plates were incubated for 24 h at 30 °C. (**C**) EOP assay of *C. crescentus* strains harboring pMT464 or pMT464-*bugA* diluted on PYE plates with or without VAN^{20}, VAN^{40} or BAC^{3}. Plates were incubated for 2 days at 30 °C. (**D**) *C. crescentus* Δ*chvT* Δ*exbD bugA*::Tn cells harboring pMT464, pMT464-*bugA*, pMT464-$bugA^{Bs}$ or other TBDR expression plasmids including pMT464-*CCNA_00171*, pMT464-*CCNA_00185*, pMT464-*CCNA_00214*, pMT464-*CCNA_00486* or pMT464-*CCNA_01194* were plated on PYE with KAN^{10}, $KAN^{10} + VAN^{10}$ or $KAN^{10} + BAC^{3}$. Plates were incubated for 3 days at 30 °C. (**E**) *WT*, Δ*bla* and derivatives expressing either an NDM1 or a Bla fusion proteins harboring the N-terminal signal sequence of YjbI in lieu of their native signal sequences, were streaked on agar plates containing doripenem (3 µg/mL) and grown for 48 h at 30 °C. The fusion proteins were expressed from the *xylX* locus using the *E. coli* phage T5 promoter. (**F**) Complementation analysis of *WT* and mutant (*mut*) YjbI expressed from P_{xyl} at the *xylX* locus in *yjbI*::Tn cells. Cells were streaked on plates containing 0.03% xylose (Xyl) and bacitracin (2.5 µg/mL, $BAC^{2.5}$) or nalidixic acid (10 µg/mL, NAL^{10}). Pates were incubated for 3 days at 30 °C. The mutant protein harbors the K284A/K287A double mutation and lacks the terminal lysine.

© The Author(s)
